# Vision-related quality of life and associated factors in individuals with vision impairment

**DOI:** 10.4102/phcfm.v17i1.4765

**Published:** 2025-02-28

**Authors:** Modjadji M. Leshabane, Nishanee Rampersad, Khathutshelo P. Mashige

**Affiliations:** 1 Discipline of Optometry, School of Health Sciences, University of KwaZulu-Natal, Durban, South Africa

**Keywords:** vision impairment, quality of life, vision-related quality of life, National Eye Institute Visual Functioning Questionnaire-39, comprehensive eye care services

## Abstract

**Background:**

Vision impairment (VI) limits the ability of affected individuals to achieve optimal functioning and impacts their quality of life.

**Aim:**

This study assesses the vision-related quality of life (VRQOL) and its associated factors in individuals with VI.

**Setting:**

The study was conducted at selected public hospitals in Limpopo province, South Africa.

**Methods:**

A cross-sectional, quantitative study was conducted between January and August 2023 across 29 public hospitals, utilising a semi-structured questionnaire. Data obtained from the participants’ responses were analysed to assess their VRQOL.

**Results:**

The study sample consisted of 454 participants, 57.0% female. The participants’ ages ranged from 18 to 82 years. The mean composite score was 22.50 ±13.01 (range: 4.0–55.7). Factors associated with increased risk of poorer VRQOL included illiteracy (*B*: –10.32; *p* < 0.001), primary level education (*B*: –6.81; *p* = 0.015) and male gender (*B*: –3.80; *p* = 0.003). Regarding VI severity and aetiology, blindness (*B*: –21.35; *p* < 0.001), cataracts (*B*: –2.98; *p* = 0.015) and corneal diseases (*B*: –6.85; *p* = 0.005) were associated with risk of poor VRQOL. Conversely, employment (*B*: 12.76; *p* < 0.001) and refractive error (*B*: 11.84; *p* < 0.001) were associated with improved VRQOL outcomes.

**Conclusion:**

The VRQOL among individuals with VI attending public hospitals in Limpopo province is relatively low. The main causes of VI were largely preventable or correctable, indicating the need to strengthen comprehensive eye care services. Enhancing these services may significantly improve the quality of life for individuals with VI in the province.

**Contribution:**

The study fills the knowledge gap regarding the impact of VI on VRQOL in individuals seeking care at public hospitals in Limpopo province.

## Introduction

Vision impairment (VI) is a major cause of disability globally and contributes to a substantial public health challenge.^1,2^ Approximately 295 million individuals worldwide experience moderate to severe VI (MSVI), while an estimated 43 million individuals are blind because of various causes.^3^ The prevalence of distance VI is approximately four times higher in low- and middle-income regions compared with high-income regions.^1^ In South Africa, VI accounts for 9.9% of all disabilities, making it the largest disability group in the country.^4^ The leading causes of VI globally are largely avoidable, treatable and/or preventable.^1,5,6^ Vision impairment negatively impacts developmental growth and is associated with diminished physical health, psycho-social well-being, economic participation and educational outcomes, ultimately resulting in a reduced quality of life.^1,7^

Vision-related quality of life (VRQOL) refers to an individual’s overall satisfaction with living conditions, functioning and how VI affects daily life.^2,8^ Comprehensive eye care services can significantly improve the quality of life of individuals with VI, enabling their full participation in social, economic, political and cultural aspects of life.^1,3,9,10,11^ The experiences of individuals with VI vary depending on factors such as age, the onset of the impairment, socio-economic status, literacy level and the availability and accessibility of comprehensive VI services.^1,2,7,12^ Vision impairment impacts individuals by causing difficulties in performing activities of daily living, increased morbidity and risk of falls, a higher risk of depression, poor educational outcomes, social isolation, reduced workplace productivity, increased dependency on care and a greater demand for health care services.^1,3,7,13,14^ Additionally, VI places significant financial burdens on affected individuals, their families and communities.^1,3,5^

The prevalence of VI in Limpopo province is relatively high, and the provision of comprehensive VI services is severely constrained in public hospitals of the region.^15,16,17,18^ The majority of the population in Limpopo province resides in rural areas and relies on public hospitals for eye care services.^19,20^ Despite these challenges, there is a paucity of research assessing VRQOL among individuals with VI in the province. This study aimed to assess the VRQOL and identify its associated factors in individuals with VI attending public hospitals in Limpopo province. Such data are crucial for assessing the impact of VI on quality of life and other visual function domains in this population. These findings will provide essential insights for policymakers, eye care personnel and Department of Health authorities, enabling informed decisions regarding planning, resource allocation and VI management. Ultimately, these efforts will improve the quality of life for affected individuals and their families in Limpopo province and serve as a foundation for future research.

## Research methods and design

### Study design

The study utilised a descriptive, quantitative, cross-sectional design to assess VRQOL and identify its associated factors in individuals with VI attending public hospitals in Limpopo province, South Africa.

### Study site and sampling

The study was conducted at selected public hospitals delivering eye care services in Limpopo province, South Africa. Between January and September 2023, 37 public hospitals in the province provided eye care services. These included 30 primary-level hospitals, five secondary-level hospitals and two tertiary-level hospitals. The services offered were relatively uniform across the hospitals in terms of patient screening, assessment, diagnosis and disease management. However, ophthalmology services were available exclusively at three public hospitals. Data were collected from 29 out of the 37 public hospitals, representing approximately 80% of the total public hospitals providing eye care services in the province. Because of the limited number of secondary and tertiary hospitals, a saturated sampling approach was applied for these levels, including those offering ophthalmology services. For the selection of primary hospitals, simple random sampling was employed to select the 22 hospitals across the various districts, ensuring each primary hospital had an equal chance of inclusion. Convenience sampling was used to recruit participants from all the selected public hospitals included in this study. Patients accessing eye care services were either self-referred or referred by outreach optometrists from district clinics and schools, or by medical doctors and other health care practitioners within the province.

### Study population

The study population consisted of individuals aged 18 years and older who had been diagnosed with VI (presenting visual acuity less than 6/18 to no light perception) for at least 3 months and were presenting for eye care services at public hospitals in Limpopo province.

### Sample size

The sample size was calculated using the following formula in equation 1:

$$n=\frac{z^{2}pq}{d^{2}}$$ [Eqn 1]

where:

*n* = sample size;

*z* = the upper point of the standard normal distribution, which is 1.96 constant when using a 95% confidence interval (CI);

*d* = the clinically acceptable margin of error of 5% (0.05);

*p* = the expected prevalence and *q* = 1–*p*.^21^

Taking into consideration the highest reported prevalence of VI found in a review of the literature as 41.3% (0.413) and *d* = 0.05 (the absolute precision, taken as 0.05), the sample size was determined in equation 2:

$$\begin{array}{l} n=\frac{{\left(1.96\right)}^{2}\times 0.413(1-0.413)}{{\left(0.05\right)}^{2}}\\ =\frac{3.8416\times 0.413\times 0.587}{0.0025}\\ =373 \end{array}$$ [Eqn 2]

To account for potential attrition, the required minimum sample size was adjusted by increasing it by 10%, resulting in a total of 411 participants.

### Inclusion and exclusion criteria

Participants aged 18 years and older, who had been diagnosed with VI for a minimum duration of 3 months and provided consent, were included in the study. The age criterion was established to ensure that participants had the cognitive capacity to comprehend and respond accurately during the interviews. The 3-month post-diagnosis period was selected under the assumption that participants had received adequate therapeutic interventions and had been fully informed of their diagnosis and prognosis. Each participant was sampled once, with follow-up visits and reviews excluded to prevent duplication, and individual participants were assigned unique codes for verification. Exclusion criteria included refusal to provide consent, participants under the age of 18 years, those unable to communicate and caregivers.

### Data collection

Data were collected using an interviewer-administered National Eye Institute Visual Functioning Questionnaire-39 (NEI VFQ-39), an internationally validated tool designed to assess the quality of life in patients with VI.^22^ This instrument has been widely employed in clinical research, with its reliability and validity established.^23^ The first section of the questionnaire comprised 12 questions related to demographic information. The NEI VFQ questionnaire included 39 items distributed across 12 sub-scales, assessing general health, general vision, ocular pain, near activities, distance activities, social functioning, mental health, role difficulties, dependency, driving, colour vision and peripheral vision, using a Likert scale. For this study, the questionnaire was translated into Sepedi, Tshivenda and Xitsonga, which are the predominant languages in Limpopo province to ensure participants could be interviewed in their preferred language. To ensure veracity, the questionnaires were back-translated into English. Participants who used glasses or contact lenses for specific tasks were instructed to respond to all questions as if they were wearing their corrective lenses. The same version of the questionnaire was administered uniformly across all study sites.

### Data analysis

The data were manually collected and entered into Microsoft Excel, after which they were analysed using the Statistical Package for Social Sciences (IBM SPSS version 29). Before analysis, the scoring of the NEI VFQ-39 was performed according to the guidelines outlined in the NEI VFQ scoring algorithm manual.^24^ Initially, the raw numeric values from the questionnaire were re-coded following the specified scoring rules. Each item was scored such that higher scores corresponded to better functioning. The re-coded scores were subsequently transformed to a standardised scale from 0 to 100, where 0 indicated the lowest possible score and 100 indicated the highest. The items within each sub-scale were then averaged to generate 12 sub-scale scores. Missing data were excluded from the calculation of the sub-scale scores. Sub-scale scores were computed for each sub-scale where at least one item was answered, representing the mean of all items within that sub-scale. The overall composite score was derived by calculating the average of the vision-targeted sub-scale scores, excluding the general health rating item. To maintain consistency across studies, all optional items pertinent to each sub-scale were incorporated into the analysis.^24^

Descriptive statistics, including frequencies, percentages, means, standard deviation (s.d.), medians and interquartile range (IQR), were employed to summarise categorical variables such as age, gender and severity of VI. The normality of the data was evaluated using the Kolmogorov–Smirnov test. Comparisons between risk factors and the outcome of interest were explored by conducting independent sample *t*-tests and analysis of variance (ANOVA). The association between socio-demographic variables and causes of VI with VRQOL were assessed using bivariate and multivariate linear regression analysis. All risk factors clinically and statistically significant with the outcome of interest were included in a multivariate model to determine the outcome variable independent factors. This approach was previously recommended in clinical research studies.^25,26^ A probability value of less than 0.05 was considered statistically significant.

### Ethical considerations

Ethical clearance to conduct this study was obtained from the University of Kwazulu-Natal Humanities and Social Science Research Ethics Committee (No. HSSREC/00004472/2022). Subsequently, gatekeeper permission was obtained from the Limpopo Provincial Department of Health (No. LP_2022-12-004) as well as from Pietersburg and Mankweng hospitals, permitting the use of their facilities for data collection. To maintain participant anonymity, unique identification codes were assigned to each participant.

## Results

### Demographic characteristics

The study sample consisted of 454 participants, with a higher proportion of females (*n* = 259, 57.0%) compared with males (*n* = 195, 43.0%). The ages of the participants ranged from 18 to 82 years, with a median of 69 (IQR: 60–78). The majority were aged 65 years and older (*n* = 292, 64.3%), followed by those aged 50–64 years (*n* = 101, 22.2%) and those aged 18–49 years (*n* = 61, 13.4%). In terms of educational attainment, most participants had completed primary-level education (*n* = 204, 44.9%), while a substantial proportion was illiterate (*n* = 129, 28.4%). A smaller group had completed secondary education (*n* = 110, 24.2%) and a minority had obtained post-matric qualifications (*n* = 11, 2.4%). The sample was predominantly African (*n* = 453, 99.8%). For workforce participation, (*n* = 206, 45.4%) had never been employed, (*n* = 209, 46.0%) had a previous employment history, (*n* = 26, 5.7%) were active in the workforce and (*n* = 13, 2.9%) had discontinued employment because of their VI.

### Clinical characteristics of participants

The majority of participants (*n* = 331, 73%) reported having chronic illnesses, with (*n* = 152, 33.5%) of these individuals presenting with two or more chronic conditions. Over half of the participants (*n* = 245, 54.0%) were diagnosed with MSVI, while (*n* = 209, 46.0%) were classified as blind. The duration of VI among participants varied: (*n* = 147, 32.4%) had experienced VI for 3–5 years, (*n* = 130, 28.6%) for 6–9 years, (*n* = 102, 22.5%) for 2 years or less and (*n* = 75, 16.5%) for 10 years or more. The leading causes of VI were cataracts (*n* = 192, 42.3%), glaucoma (*n* = 169, 37.2%), refractive error (*n* = 46, 10.1%), corneal diseases (*n* = 17, 3.7%) and retinal diseases (*n* = 30, 6.6%).

### Vision-related quality of life among participants

Table 1 displays the mean, s.d. and range of scores for the various sub-scales of the NEI VFQ-39, along with the composite score for all the participants. The mean (s.d.) scores across individual sub-scales varied, with role difficulties registering the lowest at 12.97 (15.10) and ocular pain the highest at 81.09 (19.03). Several sub-scales, including general vision, near activities, distance activities, social functioning, mental health, role difficulties, dependency, colour vision and peripheral vision, demonstrated mean scores below 20.00. The overall mean (s.d.) composite score was 22.50 (13.01). The sub-scale ‘Driving’ was consistently an outliner and non-significant in the findings because of the small sample of participants driving from the study sample.

**TABLE 1:** Mean scores for the different sub-scales in the National Eye Institute Visual Functioning Questionnaire-39 (*N* = 454).

Scale name	Score		Range
	Mean	s.d.	
Composite score	22.50	13.01	4.0–55.7
General health	38.08	16.33	15.0–95.0
General vision	14.98	12.64	0–45.0
Ocular pain	81.09	19.03	0–100.0
Near activities	14.88	14.48	0–50.0
Distance activities	13.29	14.26	0–50.0
Social functioning	16.91	17.16	0–66.7
Mental health	15.68	12.31	0–75.0
Role difficulties	12.97	15.10	0–62.5
Dependency	18.74	20.37	0–75.0
Driving	24.49†	11.20	8.3–41.7
Colour vision	19.00	18.08	0–75.0
Peripheral vision	17.95	17.18	0–50.0

s.d., standard deviation.

† , *n* = 17.

### Vision-related quality of life comparison based on gender

Table 2 presents a comprehensive comparison of the mean (s.d.) scores between male and female participants across the sub-scale items of the NEI VFQ-39. The composite score mean (s.d.) was 22.57 (13.59) for females and 22.41 (12.23) for males. Sub-scale scores for males ranged from 12.31 (14.15) for role difficulties to 81.54 (18.80) for ocular pain, whereas scores for females ranged from 13.34 (14.64) for distance activities to 80.74 (19.23) for ocular pain. Statistically significant differences (*p* < 0.05) between genders were observed for the composite score, role difficulties, colour vision and peripheral vision sub-scales (Table 2).

**TABLE 2:** Mean (standard deviation) scores in the different sub-scales based on gender.

Scale name	Sub-score				Independent *t*-test (*p*)
	Males (*n* = 195)		Females (*n* = 259)		
	Mean	s.d.	Mean	s.d.	
Composite score	22.41	12.23	22.57	13.59	0.019
General health	41.54	16.81	35.47	15.49	0.299
General vision	15.08	12.49	14.90	12.77	0.555
Ocular pain	81.54	18.80	80.74	19.23	0.613
Near activities	14.91	13.90	14.85	14.92	0.070
Distance activities	13.22	13.78	13.34	14.64	0.119
Social functioning	16.80	16.55	17.00	17.64	0.077
Mental health	14.36	11.65	16.80	12.70	0.287
Role difficulties	12.31	14.15	13.47	15.80	0.027
Dependency	19.50	19.84	18.18	20.79	0.296
Driving	23.94†	11.34	33.30‡	-	-
Colour vision	18.97	17.00	19.02	18.88	0.011
Peripheral vision	18.46	16.42	17.57	17.75	0.048

s.d., standard deviation.

† , *n* = 16;

‡ , *n* = 1.

### Vision-related quality of life based on age

Table 3 presents the mean (s.d.) scores for each sub-scale, stratified by age group. The composite scores for participants aged 18–49 years, 50–64 years and 65 years and older were 24.43 (11.87), 23.80 (13.46) and 21.65 (13.04), respectively. Among participants aged 18–49 years, the mean scores ranged from 14.10 (14.39) for the mental health subscale to 78.89 (23.00) for the ocular pain subscale. In the 50–64 age group, scores ranged from 14.11 (14.96) for role difficulties to 81.19 (17.82) for ocular pain. Participants aged 65 years and older exhibited scores ranging from 11.84 (13.80) for distance activities to 81.51 (18.55) for ocular pain. A significant difference (*p* < 0.05) was found for the general health, distance activities and dependency subscales among the three age categories (Table 3).

**TABLE 3:** Mean (standard deviation) scores in the different sub-scales stratified by age.

Scale name	Sub-score						ANOVA test (*p*)
	18–49 years (*n* = 61)		50–64 years (*n* = 101)		≥ 65 years (*n* = 292)		
	Mean	s.d.	Mean	s.d.	Mean	s.d.	
Composite score	24.43	11.87	23.80	13.46	21.65	13.04	0.165
General health	48.77	17.23	41.93	16.92	34.51	14.62	< 0.001
General vision	15.90	12.23	16.19	12.51	14.37	12.76	0.381
Ocular pain	78.89	23.00	81.19	17.82	81.51	18.55	0.621
Near activities	17.46	12.83	16.40	14.91	13.81	14.58	0.099
Distance activities	16.09	14.40	15.79	15.01	11.84	13.80	0.014
Social functioning	18.85	15.73	19.23	17.63	15.70	17.22	0.130
Mental health	14.10	14.39	15.05	11.74	16.23	12.03	0.395
Role difficulties	14.35	13.32	14.11	14.96	12.29	15.50	0.432
Dependency	27.37	23.40	19.88	20.11	16.55	19.33	< 0.001
Driving	24.97†	14.43	19.43‡	12.72	25.75§	10.84	0.713
Colour vision	21.31	15.70	21.04	18.95	17.81	18.18	0.169
Peripheral vision	20.49	14.80	19.55	18.24	16.87	17.22	0.185

s.d., standard deviation; ANOVA, analysis of variance.

† , *n* = 3;

‡ , *n* = 3;

§ , *n* = 11.

### Vision-related quality of life based on level of vision impairment

Table 4 provides a detailed comparison of mean (s.d.) scores in the different sub-scales in the NEI VFQ-39 in the two VI groups. The mean (s.d.) composite score for 32.32 (9.37) for individuals with MSVI and 10.98 (4.31) for those classified as blind. Sub-scale scores for the MSVI group ranged from 20.51 (11.94) for mental health to 80.15 (19.39) for ocular pain. In the blindness group, sub-scale scores varied from 0.54 (3.48) for role difficulties to 82.18 (18.57) for ocular pain. A statistically significant difference (*p* < 0.05) was found between the MSVI and blindness groups for the composite score and the sub-scales of general vision, near activities, distance activities, social functioning, role difficulties, dependency, colour vision and peripheral vision.

**TABLE 4:** Mean (standard deviation) scores in the different sub-scales based on the level of vision impairment.

Scale name	Sub-score				Independent *t*-test (*p*)
	MSVI (*n* = 245)		Blindness (*n* = 209)		
	Mean	s.d.	Mean	s.d.	
Composite score	32.32	9.37	10.98	4.31	< 0.001
General health	43.26	15.47	32.00	15.21	5.029
General vision	24.80	8.00	3.47	5.12	< 0.001
Ocular pain	80.15	19.39	82.18	18.57	0.616
Near activities	25.86	10.22	2.01	5.04	< 0.001
Distance activities	23.91	11.13	0.83	2.92	< 0.001
Social functioning	29.83	12.38	1.75	5.86	< 0.001
Mental health	20.51	11.94	10.02	10.15	0.371
Role difficulties	23.58	12.96	0.54	3.48	< 0.001
Dependency	33.41	16.52	1.56	5.84	< 0.001
Driving	24.49†	11.21	0^a^-	-	-
Colour vision	31.84	13.26	3.95	9.14	< 0.001
Peripheral vision	30.20	12.44	3.59	8.79	< 0.001

Note:

a , *t* cannot be computed because at least one of the groups is empty.

s.d., standard deviation; MSVI, moderate to severe VI.

† , *n* = 17.

### Vision-related quality of life comparison based on literacy level

Table 5 presents the mean (s.d.) scores of participants, categorised by literacy level. Participants with post-matric qualifications exhibited the highest composite score, 29.62 (9.28), followed by those with secondary education at 24.98 (13.43), primary education at 22.82 (12.65) and illiterate participants at 19.27 (12.82). Within the illiterate group, sub-scale scores ranged from 9.61 (13.77) for distance activities to 80.04 (18.59) for ocular pain. Among participants with primary education, scores ranged from 13.38 (13.42) for distance activities to 80.88 (19.12) for ocular pain. For those with secondary qualifications, scores varied from 14.73 (13.27) for mental health to 82.39 (19.40) for ocular pain. In participants with post-matric qualifications, the scores ranged from 15.91 (15.14) for role difficulties to 91.36 (12.38) for general health. Significant differences (*p* < 0.05) were observed across the four literacy levels in the sub-scales of general health, general vision, near activities, distance activities, social functioning, dependency, colour vision and peripheral vision.

**TABLE 5:** Mean (standard deviation) scores in the different sub-scores based on literacy level.

Scale name	Sub-score								ANOVA test (*p*)
	Illiterate (*n* = 129)		Primary education (*n* = 204)		Secondary education (*n* = 110)		Post-matric (*n* = 11)		
	Mean	s.d.	Mean	s.d.	Mean	s.d.	Mean	s.d.	
Composite score	19.27	12.82	22.82	12.65	24.98	13.43	29.62	9.28	< 0.001
General health	28.82	11.95	37.28	14.18	48.09	17.15	91.36	12.38	< 0.001
General vision	12.21	13.26	15.42	11.94	16.68	12.84	22.27	10.09	0.007
Ocular pain	80.04	18.59	80.88	19.12	82.39	19.40	84.09	20.23	0.754
Near activities	11.63	14.91	15.20	13.98	17.46	14.64	21.21	9.59	0.007
Distance activities	9.61	13.77	13.38	13.42	16.84	15.53	19.16	12.03	< 0.001
Social functioning	12.73	16.91	17.36	16.66	20.08	17.79	25.76	13.15	0.002
Mental health	15.04	11.58	16.50	11.99	14.73	13.27	17.78	16.49	0.531
Role difficulties	10.18	15.07	13.54	14.72	14.89	15.56	15.91	15.14	0.074
Dependency	13.19	18.27	18.03	18.46	24.73	23.07	37.52	25.01	< 0.001
Driving	16.63†	14.43	29.15††	14.45	23.95‡	9.37	29.15‡‡	5.87	0.509
Colour vision	14.54	17.54	19.61	17.77	22.27	18.34	27.27	13.48	0.003
Peripheral vision	13.76	16.82	18.63	17.08	20.68	17.55	27.27	7.54	0.003

s.d., standard deviation; ANOVA, analysis of variance.

† , *n* = 3;

†† , *n* = 4;

‡ , *n* = 8;

‡‡ , *n* = 2.

### Vision-related quality of life based on the cause of vision impairment

Table 6 summarises the mean (s.d.) scores of participants, classified according to the underlying cause of VI. Participants with VI because of refractive error exhibited a higher composite score of 33.15 (10.79) compared to those with glaucoma 21.95 (12.98), other diseases (corneal or retinal diseases) 21.11 (11.96) and cataracts 20.77 (12.65). Among participants with cataracts, sub-scale scores ranged from 11.00 (13.67) for distance activities to 83.07 (18.26) for ocular pain. Participants with glaucoma showed sub-scale scores ranging from 12.94 (13.89) for distance activities to 79.07 (18.68) for ocular pain. For participants with refractive error, scores ranged from 23.48 (12.15) for mental health to 77.17 (22.87) for ocular pain. Those with other diseases (e.g. corneal or retinal conditions) had scores varying from 9.05 (11.72) for role difficulties to 84.04 (18.38) for ocular pain. A significant difference (*p* < 0.05) was found between the causes of VI and the sub-scales of general vision, near activities, distance activities, social functioning, mental health, role difficulties, dependency, colour vision and peripheral vision (Table 6).

**TABLE 6:** Mean (standard deviation) scores in the different sub-scores based on the cause of vision impairment.

Scale name	Sub-score								ANOVA test (*p*)
	Cataract (*n* = 192)		Glaucoma (*n* = 169)		Refractive errors (*n* = 46)		Others (*n* = 47)		
	Mean	s.d.	Mean	s.d.	Mean	s.d.	Mean	s.d.	
Composite score	20.77	12.65	21.95	12.98	33.15	10.79	21.11	11.96	< 0.001
General health	37.98	15.89	36.83	16.25	39.29	15.89	41.76	18.58	0.306
General vision	13.36	12.09	14.66	12.84	25.54	9.67	12.45	11.97	< 0.001
Ocular pain	83.07	18.27	79.07	18.68	77.17	22.87	84.04	18.38	0.069
Near activities	13.15	14.49	14.38	14.11	27.00	11.92	11.86	12.49	< 0.001
Distance activities	11.01	13.67	12.94	13.89	25.21	12.61	12.20	14.14	< 0.001
Social functioning	14.30	16.46	16.64	16.91	31.35	15.24	14.37	16.18	< 0.001
Mental health	14.45	11.46	14.85	11.04	23.48	12.15	16.06	16.91	< 0.001
Role difficulties	11.67	15.51	13.09	14.63	24.05	13.43	9.05	11.72	< 0.001
Dependency	15.73	19.64	18.35	20.15	33.57	17.99	17.97	20.72	< 0.001
Driving	20.82†	11.48	22.90††	112.49	28.32‡	112.65	29.15‡‡	5.87	0.689
Colour vision	16.28	17.12	18.49	18.15	33.70	16.00	17.55	17.22	< 0.001
Peripheral vision	15.76	16.85	17.16	16.86	32.07	14.59	15.96	16.00	< 0.001

Note: Others – corneal or retinal diseases.

s.d., standard deviation; ANOVA, analysis of variance.

† , *n* = 6;

†† , *n* = 4;

‡ , *n* = 5;

‡‡ , *n* = 2.

### Factors associated with vision-related quality of life

Table 7 shows the bivariate and multivariate linear regression analysis for VRQOL. The bivariate regression analysis showed that illiterate participants (*B*: –10.32; 95% CI: –15.98 to –4.65; *p* < 0.001), and those with primary level education (*B*: –6.81; 95% CI: –12.31 to –1.31; *p* = 0.015) had significantly increased risk of poor VRQOL. Participation in the workforce (*B*: 12.76; 95% CI: 9.05 to 16.47; *p* < 0.001) was significantly associated with reduced risk of poor VRQOL. In terms of the level of VI, blindness (*B*: –21.35; 95% CI: –22.66 to –20.05; *p* < 0.001) was significantly associated with an increased risk of poor VRQOL. About the causes of VI, the presence of cataracts (*B*: –2.98; 95% CI: –5.36 to –0.59; *p* = 0.015) and corneal diseases (*B*: –6.85; 95% CI: –11.64 to –2.07; *p* = 0.005) were significantly associated with increased risk of poor VRQOL. However, the presence of refractive error (*B*: 11.84; 95% CI: 8.52 to 15.16; *p* < 0.001) was significantly associated with improved VRQOL outcomes. In the multivariate regression analysis, male gender (*B*: –2.30; 95% CI: –3.80 to –0.80; *p* = 0.003) and blindness (*B*: –20.42; 95% CI: –21.84 to –19.00; *p* < 0.001) were significantly associated with increased risk of poor VRQOL.

**TABLE 7:** Bivariate and multivariate linear regression for predictors of vision-related quality of life (*N* = 454).

Factors associated with VRQOL	Bivariate regression			Multivariate regression		
	*B*	95% CI	*P*	*B*	95% CI	*P*
**Gender**						
Female	Ref	-	-	-	-	-
Male	−0.18	−2.56 to 2.20	0.882	−2.30	−3.80 to −0.80	0.003
**Age (years)**						
≥ 65	Ref	-	-	-	-	-
18–49	2.77	−0.54 to 6.07	0.101	−2.12	−4.79 to 0.55	0.120
50–64	2.13	−0.87 to 5.14	0.164	−1.33	−3.12 to 0.46	0.144
**Literacy level**						
Primary education	−6.81	−12.31 to −1.31	0.015	−3.52	−8.20 to 1.152	0.140
Secondary education	−4.65	−10.43 to 1.14	0.116	−2.079	−6.65 to 2.49	0.373
Post matric	Ref	-	-	-	-	-
Illiterate	−10.32	−15.98 to −4.65	< 0.001	−4.54	−9.35 to 0.27	0.064
**Workforce participation**						
No	Ref	-	-	-	-	-
Yes	12.76	9.05 to 16.47	< 0.001	2.77	−1.04 to 6.59	0.154
**Hypertension**						
No	Ref	-	-	-	-	-
Yes	−0.78	−3.21 to 1.64	0.525	0.03	−1.50 to 1.56	0.967
**Diabetes mellitus**						
No	Ref	-	-	-	-	-
Yes	0.11	−2.39 to 2.61	0.930	−0.72	−2.45 to 1.02	0.420
**Retroviral diseases**						
No	Ref	-	-	-	-	-
Yes	3.30	−1.38 to 7.98	0.167	0.40	−2.30 to 3.09	0.774
**Level of VI**						
MSVI	Ref	-	-	-	-	-
Blindness	−21.35	−22.66 to −20.05	< 0.001	−20.42	−21.836 to −19.00	< 0.001
**Duration of VI (years)**						
≥ 10	Ref	-	-	-	-	-
0–2	3.39	−0.38 to 7.16	0.078	1.12	−1.373 to 3.61	0.378
3–5	3.11	−0.39 to 6.61	0.082	0.46	−1.72 to 2.64	0.681
6–9	2.86	−0.78 to 6.61	0.123	−1.10	−3.07 to 0.88	0.277
**Cataract**						
No	Ref	-	-	-	-	-
Yes	−2.98	−5.36 to −0.59	0.015	−2.57	−8.40 to 3.26	0.387
**Refractive error**						
No	Ref	-	-	-	-	-
Yes	11.84	8.52 to 15.16	< 0.001	2.09	−4.43 to 8.61	0.529
**Glaucoma**						
No	Ref	-	-	-	-	-
Yes	−0.89	−3.35 to 1.58	0.482	−1.21	−7.19 to 4.77	0.691
**Corneal anomalies**						
No	Ref	-	-	-	-	-
Yes	−6.85	−11.64 to −2.07	0.005	−1.72	−7.72 to 4.29	0.574
**Retinal anomalies**						
No	Ref	-	-	-	-	-
Yes	1.30	−3.15 to 5.75	0.567	−1.43	−7.03 to 4.16	0.616

VRQOL, vision-related quality of life; VI, vision impairment; CI, confidence interval; MSVI, moderate to severe vision impairment; B, regression slope.

## Discussion

Vision impairment limits affected individuals from achieving their full functional potential, hinders educational attainment, reduces productivity in employment and compromises independence in daily life. This study aimed to assess the VRQOL and its associated factors in individuals with VI attending public hospitals in Limpopo province.

Participants in this study exhibited relatively low scores across all subscales, including the composite score (Table 1), indicating poor functioning in multiple domains of VRQOL. The poor VRQOL may be attributed to factors such as the characteristics of the sample population, the onset of VI, access to VI-related services and socio-demographic factors known to influence VRQOL.^1,2,7,12^ Specifically, the sample comprised individuals who experienced varying degrees of functional vision limitations for a minimum of 3 months. These findings align with previous research,^2,12,27,28^ which similarly demonstrated that VI contributes to a reduction in VRQOL. The majority of participants in this study were unemployed, resided in rural areas and exhibited low literacy levels. These socio-demographic factors have been associated with poor VRQOL in individuals with VI.^2,27,29^ Furthermore, most participants had acquired their VI, which may necessitate adaptation and acceptance, with the development of new coping mechanisms and access to rehabilitation services to effectively function in their daily lives.^1,3,5,30^ However, evidence suggests that the provision of VI services in Limpopo province remains inadequate, with large portions of the region currently underserved.^15,16,18,31^

The bivariate regression analysis indicated that participants with low literacy levels and those diagnosed with blindness were significantly associated with an increased risk of poorer VRQOL. The low literacy levels may have contributed to a lack of awareness, reduced health-seeking behaviours and limited utilisation of eye care services among these individuals, as compared to their counterparts.^1,2,12,32^ This could be confirmed by the significant differences in the mean scores for almost all sub-scales, with poorer scores reported among illiterate participants than those with primary education, secondary education or post-matric respectively (Table 5). The poorer VRQOL observed in participants with blindness was anticipated, as a significantly reduced level of VI typically leads to greater restrictions on functional ability and participation in daily activities.^1,3,5^ This was corroborated by the markedly low mean score for role difficulties among these individuals (Table 4). The findings of this study align with those from previous research,^2,27,29,33,34,35^ which have consistently demonstrated that the severity of VI is associated with a diminished quality of life.

In relation to the cause of VI, the presence of cataracts and corneal diseases exhibited a significant association with diminished VRQOL. This could be attributed to differences in the onset, progression and severity of these conditions. For example, individuals with VI because of age-related cataracts may experience gradual and substantial vision deterioration as the condition advances, which impairs their ability to perform daily tasks and consequently impacts their quality of life. This could be associated with the lower mean scores for distance activities and role difficulties among participants with VI because of cataracts (Table 6). Furthermore, the severity and/or complications associated with corneal diseases, compounded by limited access to effective therapeutic interventions, may result in chronic VI, increasing dependency among affected individuals. The reduced risk of poor VRQOL because of refractive error may be because people with refractive errors tend to adjust to their working distances compared to other visual disabling conditions.

Moreover, participation in the workforce was significantly associated with improved VRQOL outcomes. This finding is consistent with reports,^27,33^ which demonstrated an improved quality of life among employed individuals with VI in comparison to unemployed persons. Financial constraints among individuals with VI may further influence their eye care-seeking behaviours, leading to delayed clinical interventions, which could result in complications of treatable eye conditions and substantially decrease the quality of life for those affected.^1^

Although the bivariate and multivariate analysis showed no significant association between age and VRQOL, increasing age was associated with an increased risk of poor VRQOL, with participants aged 18–49 years old exhibiting reduced risk of poor VRQOL compared to participants aged 50–64 years and 65 years and older, respectively (Table 7). This could be attributed to the sample population in this study. For instance, almost 65% of participants from this study were 65 years and older and at increased risk of developing VI because of age-related eye diseases including cataracts and glaucoma, which significantly reduce the quality of life of affected individuals.^1,5^ Interestingly, participants aged 18–49 years old showed low mean scores for mental health and role difficulties (Table 3). This demonstrates the effect of VI on the psychological well-being of affected individuals and its impact on daily activities.

The multivariate regression analysis revealed that male gender and blindness were significantly associated with an increased risk of poorer VRQOL. The lower VRQOL scores observed among males in this study may be attributed to their reduced performance in the ‘role difficulties’ subscale, indicating functional limitations (Table 2). This finding could be related to difficulties in adopting new coping mechanisms and concerns over loss of independence. Similar results were reported by Jammal et al.,^12^ who found that males were at a heightened risk for poor VRQOL despite the higher prevalence of VI among females.^1,5^ These findings suggest a need for more targeted interventions and/or support programmes, particularly for males and those with blindness, to ensure that they are better supported to achieve optimal quality of life.

Despite these findings, the experience and adaptation to living with VI are influenced by factors such as the onset and severity of the condition, socio-economic status, psycho-social well-being and the availability and accessibility of preventive, treatment and vision rehabilitation services.^1,3,5^ The majority of VI causes identified in this study are reversible through medical, surgical or optical interventions. This highlights the need to strengthen and implement comprehensive VI services to reduce the burden of VI among individuals utilising these public hospitals.

### Strength and limitations

The strengths of this study include the use of the internationally validated NEI VFQ-39 questionnaire, which enhances the comparability of findings with other research. The questionnaire was translated into the predominant languages spoken in the Limpopo province, ensuring that participants were interviewed in their preferred language. Although this process was essential, we acknowledge that certain words may not have been fully translatable in the local context. A limitation of the study is its hospital-based design, which may limit the generalisability of the results. Additionally, recall bias may have affected the findings, as certain questions relied on participants’ recollections of past experiences. The study included a limited number of participants who engaged in driving, likely because of their recruitment from public hospitals located in a socio-economically disadvantaged province, coupled with VIs that restricted their eligibility for driving. Consequently, the findings related to driving should be interpreted with caution. Despite these limitations, this study provides valuable insights for policymakers, health authorities and eye care professionals, facilitating the effective planning of comprehensive eye care services. It also offers a foundation for future research.

## Conclusion

The VRQOL in individuals with VI attending public hospitals in Limpopo province was found to be suboptimal. The results indicate that VI significantly impacts psychosocial functioning, limits individuals’ participation in daily activities and leads to a diminished overall quality of life. These findings suggest the need to prioritise the reduction of avoidable VI and improve access to comprehensive eye care services, which are essential for enhancing the full participation and functioning of affected individuals in their daily lives.
